# NLRP3 Inflammasome Inhibition by Xuanfei Baidu Decoction Attenuates Pulmonary Inflammation and Collagen Deposition in Silicosis

**DOI:** 10.3390/ph19020253

**Published:** 2026-02-01

**Authors:** Qianru Zhao, Junhong Wang, Ziwei Yan, Tao Liu, Lin Ma, Jing Qian, Yu Wang, Rui Shao

**Affiliations:** 1 Institute of Traditional Chinese Medicine, Tianjin University of Traditional Chinese Medicine, Tianjin 301617, China; 2 State Key Laboratory of Chinese Medicine Modernization, Tianjin University of Traditional Chinese Medicine, Tianjin 301617, China; 3 School of Medical Technology, Tianjin University of Traditional Chinese Medicine, Tianjin 300193, China; 4 Pharmaceutical Informatics Institute, College of Pharmaceutical Sciences, Zhejiang University, Hangzhou 310058, China; 5 School of Integrative Medicine, Tianjin University of Traditional Chinese Medicine, Tianjin 301617, China; 6 Haihe Laboratory of Modern Chinese Medicine, Tianjin University of Traditional Chinese Medicine, Tianjin 301617, China

**Keywords:** Xuanfei Baidu decoction, silicosis, macrophages, NLRP3 inflammasome, EMT

## Abstract

**Background/Objectives**: Silicosis is a chronic disease caused by long-term exposure to high levels of silica dust, which leads to extensive nodular fibrosis in the lungs. The disease is currently a serious occupational health hazard globally. Xuanfei Baidu decoction (XFBD) is a mature Chinese herbal medicine in China that has shown anti-inflammatory and anti-fibrotic effects in mouse experiments, making it a promising candidate for addressing the persistent inflammation and fibrosis in silicosis. **Methods**: Silicosis was induced in male C57BL/6J mice using crystalline silica (CS). XFBD’s early anti-inflammatory role was verified in vitro in peritoneal macrophages (PMs) and in vivo in silicosis mice, while its late anti-collagen deposition and anti-fibrotic activities were further investigated. **Results**: In vitro, XFBD effectively inhibits the activation of the NOD-like receptor thermal protein domain-associated protein 3 (NLRP3) inflammasome in CS-induced lipopolysaccharide (LPS)-primed PMs, decreases the release of inflammatory cytokines, including interleukin (IL)-1β, IL-6, and tumor necrosis factor-α (TNF-α), and modulates the phenotypic transition of macrophages from the M2 to the M1 phenotype. In vivo studies further validated that XFBD significantly downregulates the expression of NLRP3 and Cleaved-Caspase-1 proteins in the lung tissues of mice afflicted with silicosis. Additionally, XFBD enhanced pulmonary function, inhibited collagen deposition and pulmonary fibrosis in silicosis mice, and reversed epithelial–mesenchymal transition (EMT) by regulating key EMT-related proteins to slow fibrosis. **Conclusions**: The beneficial effects of XFBD on CS-induced pulmonary fibrosis can be attributed to the induction of macrophage polarization-mediated anti-inflammatory responses during the early stage of fibrotic development, as well as its anti-collagen deposition and anti-fibrotic activities during the intermediate stage of fibrotic development. This study provides preclinical evidence supporting XFBD as a promising candidate for prevention or adjunctive therapy, and its multi-target, time-phase mechanism offers a novel rationale and theoretical foundation for the development of new strategies against silicosis.

## 1. Introduction

Silicosis represents a progressive pulmonary disorder caused by prolonged occupational exposure to inhalable crystalline silica (CS), predominantly in dust-producing operations, including machining, fracturing, or pulverization processes [1,2]. Inhalation of inhalable silica particles causes minerals to be deposited in the terminal bronchi and alveoli, triggering inflammatory reactions in lung tissue and promoting fibroblast proliferation through complex pathogenic mechanisms, eventually leading to fibrosis [3]. At present, the available treatment options are severely limited, mainly aimed at alleviating symptoms, preventing disease progression, improving the overall condition, and reducing the risk of related diseases. There is still a key unmet clinical need for safe and effective drug treatment interventions to prevent the occurrence and development of this disease.

The progression of pulmonary fibrosis caused by CS is irreversible, and the impairment of lung function continues to advance [4]. The fibrotic progression induced by silicon dioxide advances over time, accompanied by specific pathological symptoms. In the acute phase, within 7 days after exposure, there is obvious macrophage and neutrophil infiltration, highlighting the intensification of the inflammatory response at this initial stage. Collagen synthesis, a key marker of pulmonary fibrosis, undergoes significant regulation during this period. Research indicates that by day 28 post-exposure, expression levels of collagen subtypes I and III are markedly elevated, reflecting the commencement of accelerated collagen deposition. The fibrotic stage represents an advanced phase in the pulmonary fibrosis model. Long-term research shows that after 28 days, the severity of pulmonary fibrosis is significantly aggravated, and lung function is significantly reduced. By day 56 of exposure, extensive confluent fibrotic nodules have formed within the lung tissue, demonstrating maturation of the fibrotic process [5]. Gradually, these pulmonary nodules merge together, annihilate the normal lung tissue, and cause progressive massive fibrosis. These findings collectively suggest that the critical period for fibrotic development predominantly occurs between the 7th and 28th days following silica exposure, a timeframe characterized by the transition from the active inflammation phase to the establishment of a mature fibrotic architecture.

The NOD-like receptor thermal protein domain-associated protein 3 (NLRP3) inflammasome is an important part of innate immunity and plays a central role in inflammatory regulation [6,7]. Emerging evidence highlights that silica dust exposure triggers NLRP3 activation at the early stage of fibrogenesis, primarily through mechanisms such as lysosomal rupture, mitochondrial dysfunction, or ionic imbalance [8,9,10]. This early activation event drives an IL-1β-mediated inflammatory cascading response and subsequent fibrosis progression. NLRP3 inflammatory body induced expression in monocytes/macrophages [11]. Furthermore, the NLRP3 inflammasome is implicated in CS-induced lung epithelial cell injury and aberrant regeneration, leading to epithelial structure destruction, excessive mucus production, and epithelial–mesenchymal transition (EMT) [6,12]. The NLRP3 inflammasome is a widely distributed cytosolic multiprotein complex, consisting of NLRP3, apoptosis-associated speck-like protein containing a caspase activation and recruitment domain (ASC), and pro-Caspase-1. Once assembled, this inflammasome drives the maturation of pro-Caspase-1 and converts pro-interleukin-1β (pro-IL-1β) and pro-IL-18 into their biologically active forms [6]. Mounting evidence in silicosis highlights the crosstalk between the NLRP3 inflammasome and programmed cell death (autophagy, apoptosis, pyroptosis)—a cascade closely linked to EMT. Silica-induced lysosomal disruption activates NLRP3-ASC complex formation, while autophagy deficiency amplifies NLRP3 activity to promote EMT. NLRP3-mediated IL-1β release regulates macrophage apoptosis via the inducible nitric oxide synthase (iNOS)–nitric oxide (NO) cascade, with tumor necrosis factor-α (TNF-α)/nuclear factor kappa-B (NF-κB) signaling fine-tuning apoptotic responses that drive epithelial–mesenchymal transition. Pyroptosis, directly triggered by NLRP3–ASC–Caspase-1 activation, cleaves gasdermin D to induce inflammatory cell death to accelerate EMT [13]. It is worth noting that the intervention strategy of targeted NLRP3 assembly or cytokine signal conduction at the precursor stage has shown the potential to delay the progression of fibrosis, highlighting the therapeutic significance of early NLRP3 regulation in CS-induced pulmonary fibrosis.

Xuanfei Baidu Decoction (XFBD), a well-known traditional Chinese herbal formula in China, is clinically beneficial for treating lung inflammation [14]. Previous studies have shown that XFBD improves mitochondrial dynamic disorders and NLRP3 inflammatory body activation in lipopolysaccharide (LPS)-induced acute lung injury and inflammation models by inhibiting NF-κB and MAPK signaling pathways [15]. Additionally, XFBD has been shown to mitigate bleomycin-induced idiopathic pulmonary fibrosis in mice by modulating lung-gut crosstalk through the IFNγ/STAT1/STAT3 signaling axis [16]. Furthermore, XFBD suppresses macrophage-mediated inflammation and bleomycin-induced pulmonary fibrosis by inhibiting the IL-6/STAT3 signaling pathway [17]. XFBD also attenuates acute lung injury and modulates inflammatory responses by suppressing macrophage-derived cytokine synthesis and preventing complement overactivation [18]. Based on the above evidence, we hypothesized that the activation of NLRP3 inflammatory small body through XFBD pharmacological regulation in the early stage of inflammation can effectively intervene in the inflammatory cascading reaction triggered by CS and hinder the subsequent fibrosis process. Elucidating the mechanisms by which XFBD confers its protective effects against CS-induced pulmonary fibrosis progression, specifically dissecting the mechanistic basis for its efficacy during the critical therapeutic window, is essential for optimizing its clinical application and warrants detailed mechanistic studies.

This study seeks to thoroughly employ in vivo and in vitro models to elucidate the dual-phase protective effects of XFBD during the early (anti-inflammatory) and advanced (anti-collagen deposition/anti-fibrosis) stages of CS-induced pulmonary fibrosis. Additionally, it aims to investigate the molecular mechanisms through which XFBD influences multiple targets, including macrophage polarization, the NLRP3 inflammasome, and EMT. The research will provide a comprehensive analysis of the multi-link mechanisms involved in XFBD intervention throughout the dynamic progression of silicosis fibrosis. Furthermore, it aims to furnish empirical evidence supporting the multi-component, multi-target, and multi-stage treatment of complex diseases using traditional Chinese medicine compound prescriptions.

## 2. Results

### 2.1. XFBD Inhibits CS-Induced NLRP3 Inflammasome Activation In Vitro

**Figure 1:**
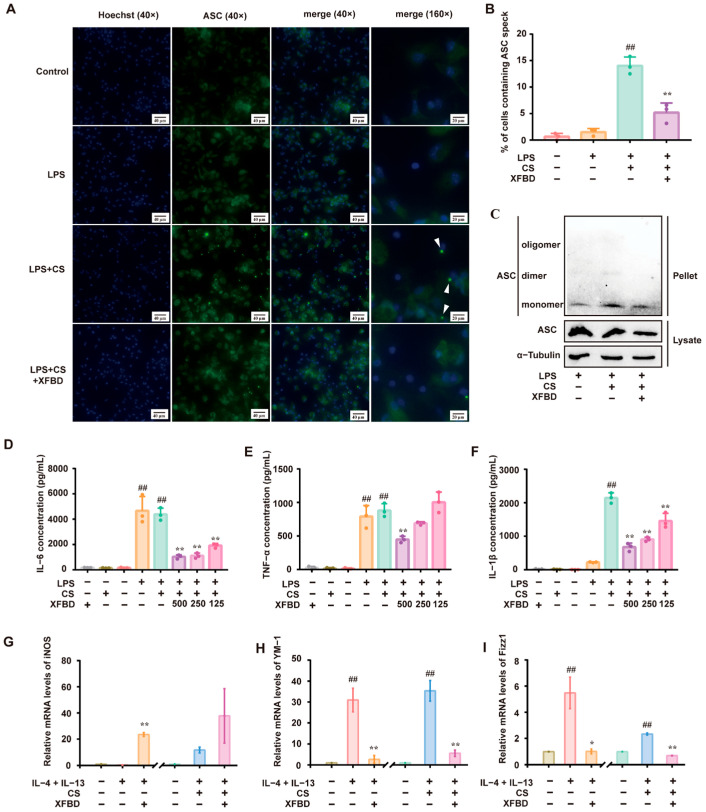
XFBD inhibits silica-related NLRP3 inflammasome activation in vitro. (**A**) Immunofluorescence of ASC (green) and Hoechst (blue) was carried out to examine ASC speck formation. (**B**) The percentage of cells containing ASC specks relative to total cells in three independent experiments is shown. (**C**) ASC oligomerization was assessed by Western blotting to investigate ASC monomers, dimers, and oligomers. The levels of IL-6 (**D**), TNF-α (**E**), and IL-1β (**F**) in the culture medium were detected by ELISA. The relative mRNA levels of an M1 polarization gene biomarker (iNOS (**G**)) and M2 polarization gene biomarkers (YM-1 (**H**), Fizz1 (**I**)) are shown. (**B**,**D**–**F**) ##: *p* < 0.01 vs. the control group (no treatment); **: *p* < 0.01 vs. the LPS+CS group.(**G**–**I**) ##: *p* < 0.01 vs. the control group (no treatment); **: *p* < 0.01 vs. the IL-4+IL-13 group or the IL-4+IL-13+CS group. Statistical analysis was performed using one-way ANOVA. Data are presented as mean ± SD.

Preliminary studies confirmed that XFBD inhibits the activation of the NLRP3 inflammatory body by inhibiting NF-κB and MAPK signaling pathways in LPS-induced acute lung injury [15]. To elucidate the direct regulatory effects of XFBD on CS-induced NLRP3 inflammasome activation, we conducted a series of experiments. Macrophages, primed with LPS, were exposed to CS to activate the NLRP3 inflammasome [19]. We assessed ASC-speck formation and ASC oligomerization as hallmarks of NLRP3 inflammasome activation [20]. Immunofluorescence staining revealed the formation of ASC specks in LPS/CS-activated peritoneal macrophages (PMs), as anticipated. XFBD remarkably reduced the number of ASC specks (Figure 1A,B). Similarly, Western blotting showed that treatment with XFBD suppressed ASC oligomerization (Figure 1C). LPS-primed PMs produced significant amounts of IL-6 and TNF-α. Subsequent stimulation by CS led to a robust release of IL-1β. Consistent with previous studies, XFBD significantly attenuated the production of IL-6 and TNF-α (Figure 1D,E) [17,21,22]. Notably, XFBD also attenuated the release of IL-1β in a dose-dependent manner (Figure 1F), collectively indicating its inhibitory effect on CS-induced NLRP3 inflammasome activation.

Building upon the confirmation that XFBD can directly inhibit the activation of the NLRP3 inflammasome, we investigated its potential impact on the inflammatory process by examining its influence on the upstream event of macrophage polarization. The polarization state of macrophages is known to be intricately linked to the activity of the NLRP3 inflammasome [23]. Macrophages can be divided into M1 and M2 types based on their functional properties. IL-4/IL-13 was used to induce macrophage M2 polarization. The expression of YM-1, Fizz1 (biomarkers of M2 polarization), and iNOS (a biomarker for M1 polarization) was detected [24]. In some of the experiments, CS was additionally added to IL-4/IL-13 since it is assumed that macrophage M2 polarization may increase silica uptake, and subsequent avid uptake of silica might render macrophages more susceptible to cell death, thereby aggravating the inflammatory cascade and exacerbating silicosis [25]. As expected, IL-4/IL-13 stimulated the expression of YM-1 and Fizz1 but was decreased by XFBD. Notably, XFBD upregulated the expression of iNOS, which indicated a transformation of macrophages from M2 to M1. Applying CS to IL-4/IL-13 resulted in higher levels of YM-1 and Fizz1. Again, XFBD alleviated the expression of YM-1 and Fizz1, along with a moderate enhancement of iNOS (Figure 1G–I). In summary, the results demonstrate that XFBD effectively inhibits CS-induced activation of the NLRP3 inflammasome and exerts a regulatory influence, promoting the phenotypic skewing of macrophages from the M2 to the M1 phenotype. Meanwhile, we confirmed that the dosage of XFBD used in this experiment had no effect on the cell viability of PM cells (Supplementary Figure S1A). These findings suggest that XFBD not only inhibits inflammasomes but also modulates the functional state of immune cells upstream, reversing M2-type polarization, which may contribute to fibrosis development.

### 2.2. XFBD Inhibits CS-Associated Macrophage Polarization and NLRP3 Inflammasome Activation In Vivo

**Figure 2:**
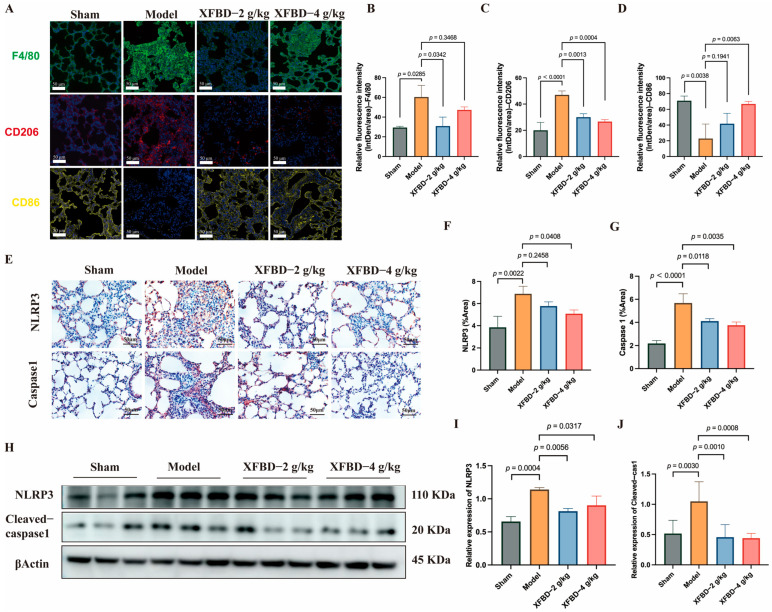
XFBD inhibits silica-associated macrophage polarization and NLRP3 inflammasome activation. Immunofluorescence imaging and quantification of F4/80, CD206, and CD86 (**A**–**D**) (*n* = 5). IHC analysis of paraffin-embedded, silica-induced, pulmonary fibrosis mice lung tissue sections using antibodies (NLRP3 and Caspase-1) on day 28 (**E**–**G**) (*n* = 5). Protein (NLRP3 and Cleaved-Caspase-1) levels in lung tissues of silica-induced pulmonary fibrosis mice were detected by Western blot (**H**–**J**) (*n* = 3). Statistical analysis was performed using one-way ANOVA. Data are presented as mean ± SD.

Building upon the initial in vitro experiments, which demonstrated that XFBD inhibits NLRP3 activation in peritoneal macrophages and regulates macrophage phenotypic transformation, this study aims to further substantiate and explore the inhibitory effects of XFBD on NLRP3 activation and macrophage polarization in a murine model of silicosis. We began by analyzing F4/80-positive macrophages in the lung tissue of mice. Immunofluorescence analysis revealed an increased presence of F4/80-positive cells in silicosis model mice, predominantly localized within fibrous nodules. Notably, treatment with XFBD resulted in a reduction of this accumulation. Additionally, our findings indicated that XFBD significantly downregulated the expression of CD206, a marker of M2 macrophage polarization, while upregulating CD86, a marker associated with M1 polarization (Figure 2A–D). The activation of the NLRP3 inflammasome promotes M1 macrophage polarization, thereby enhancing IL-1β production and exacerbating the inflammatory response [26,27]. Inhibition of the NLRP3 inflammasome has the potential to alleviate both inflammation and fibrosis [28]. We evaluated the macrophage polarization process by modulating NLRP3. IHC analysis was employed to visualize the in situ expression of NLRP3 and Caspase-1 in lung tissues. CS increased the expression of NLRP3 and Caspase-1, and XFBD dramatically reversed the elevation of NLRP3 and Caspase-1 (Figure 2E–G). Consistently, Western blotting results demonstrated that the protein levels of NLRP3 and Cleaved-Caspase-1 were elevated in the model group, which could also be reversed by XFBD treatment at a high dosage (4 g/kg) (Figure 2H–J). These results suggest that XFBD treatment can significantly inhibit CS-induced inflammasome activation in pulmonary fibrosis. No obvious pathological changes (e.g., hepatocellular necrosis, renal tubular damage) were observed in liver/kidney sections of mice in the XFBD-treated groups (4.0 g/kg and 2.0 g/kg) compared with the sham group. These findings confirm that the experimental doses of XFBD did not induce toxic damage to key metabolic organs in mice (Supplementary Figure S1B).

In vitro experiments demonstrated that XFBD effectively inhibits ASC oligomerization and IL-1β release. In vivo studies further validated that XFBD significantly downregulates the expression of NLRP3 and Cleaved-Caspase-1 proteins in the lung tissues of mice with silicosis. This evidence underscores that inhibiting NLRP3 inflammasome activation is a crucial pathway through which XFBD exerts its anti-inflammatory effects against silicosis. Additionally, XFBD has shown the capacity to regulate macrophage polarization in both in vivo and in vitro settings, indicating that XFBD may directly or indirectly attenuate the excessive activation of the NLRP3 inflammasome by correcting abnormal macrophage polarization (reducing M2), thereby decreasing the production of downstream fibrogenic factors such as IL-1β. This process potentially disrupts the “inflammation-fibrosis” cycle in the early stages of the disease.

### 2.3. XFBD Treatment Improved Lung Function in Mice with Silicosis

**Figure 3:**
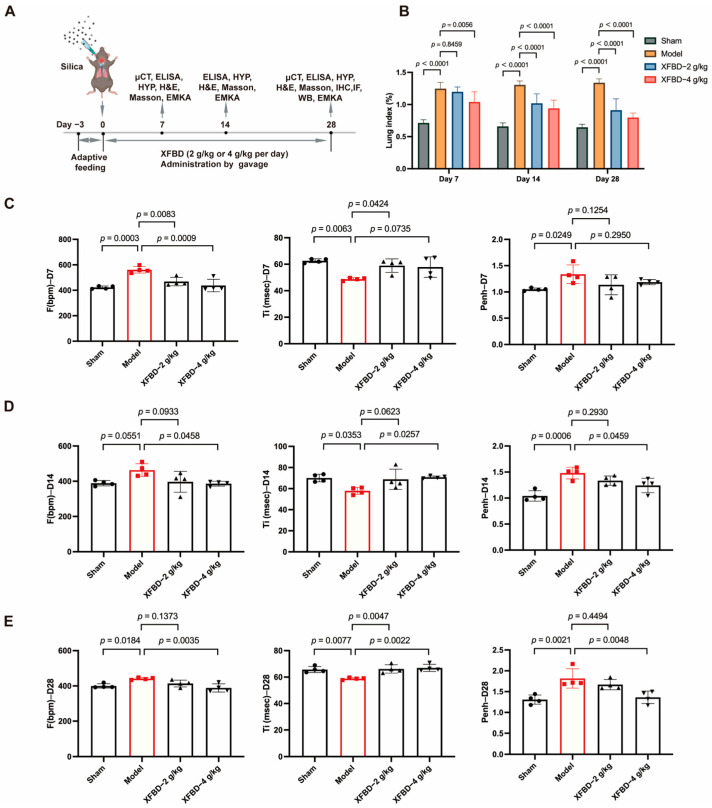
Effect of XFBD on lung function in silicosis mice induced by silica. (**A**) Experimental outline. C57BL/6 mice were given a silica suspension (50 mg/kg) into the lung once using intratracheal injection on day 0, followed by oral administration with a single dose of XFBD (2 g/kg; 4 g/kg) every day for 28 d. Mice were sacrificed on day 28. (**B**) Lung index of silicosis mice at different time points (*n* = 5). (**C**–**E**) Lung function in silicosis mice was measured at 7 d, 14 d and 28 d (*n* = 4). In the figure, circles represented the sham group, squares represented the model group, triangles represented the XFBD-2 g/kg group, and inverted triangles represented the XFBD-4 g/kg group. One-way ANOVA was used for data with only “group” as the factor; repeated measures ANOVA was applied for data involving two variables (group and time).

We have demonstrated the inhibitory effect of XFBD on NLRP3 inflammasome activation through both in vitro and in vivo silicosis models. Considering the activation of NLRP3 represents an early event in CS-induced fibrosis, we are prompted to hypothesize whether XFBD could potentially delay the progression of CS-induced fibrosis and protect against pulmonary injury by suppressing NLRP3 inflammasome activation. To investigate the effects of XFBD on improving lung function in silicosis mice, male C57BL/6J mice (8 weeks old) were administered a single intratracheal instillation of CS suspension (50 mg/kg) to induce lung injury (Figure 3A). Considering that the timeframe spanning from day 7 to day 28 post-CS exposure constitutes a pivotal phase for fibrotic progression, characterized by a pharmacological transition from an acute inflammatory response to the formation of stabilized, mature fibrotic lesions, we conducted a series of assessments in a CS-induced murine model. Specifically, we quantified the lung index and evaluated pulmonary function parameters in mice at days 7, 14, and 28 following CS administration, with the objective of determining the pharmacodynamic effects of XFBD when administered as an early therapeutic intervention for silica-induced pulmonary fibrosis. We found that, with longer exposure to CS, XFBD treatment significantly reduced the lung index compared to the model group, especially in the high dose group (Figure 3B).

To further evaluate the lung function in mice, non-invasive lung function instruments were used to assess various indicators of mice. The findings indicated that, in comparison to the sham-operated group, mice exposed to CS for 7 days demonstrated an increased respiratory frequency (F), reduced inspiratory time (Ti), and an elevated enhanced pause (Penh). Furthermore, these symptoms progressively worsened with extended CS exposure. However, concurrent administration of XFBD, particularly at high doses, significantly ameliorated the increased respiratory frequency in mice by day 7. By days 14 and 28, XFBD not only alleviated the elevated respiratory frequency but also restored the inspiratory time and enhanced pause, thereby preserving pulmonary function in the mice (Figure 3C–E).

### 2.4. XFBD Inhibited Collagen Deposition and Pulmonary Fibrosis Formation

**Figure 4:**
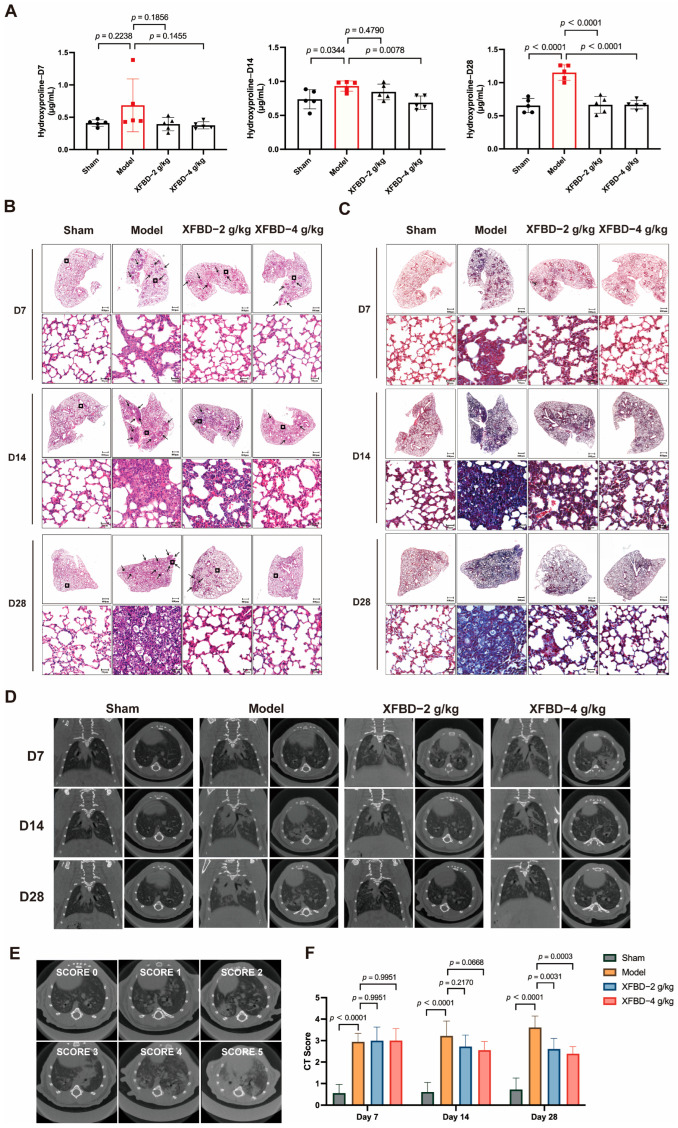
Effect of XFBD on silica-induced early inflammation and end-stage pulmonary fibrosis in mice at different time points. (**A**) The content of hydroxyproline was detected using the Hydroxyproline Assay Kit (*n* = 5). (**B**) Representative photos of HE staining in lung tissue at 7, 14, and 28 days. (*n* = 5). (**C**) Representative photos of Masson staining in mouse lung tissue at 7, 14, and 28 days. (*n* = 5). (**D**) Different visual fields were obtained from seven spinal columns (*n* = 5). (**E**) Example of scoring of the severity of consolidation on Micro CT. Consolidation was scored on a 0 to 5 scale (absent = 0, less than 5% = 1, 5–25% = 2, 25–50% = 3, 50–75% = 4, more than 75% = 5) depending on the area involved. In a silica-induced pulmonary fibrosis mouse model, the severity of imaging manifestations before and after XFBD treatment was assessed semi-quantitatively on a five-point scale. (**F**) CT Score at 7, 14, and 28 days. In the figure, circles represented the sham group, squares represented the model group, triangles represented the XFBD-2 g/kg group, and inverted triangles represented the XFBD-4 g/kg group. One-way ANOVA was used for data with only “group” as the factor; repeated measures ANOVA was applied for data involving two variables (group and time).

Collagen deposition is a critical hallmark of pulmonary fibrosis. Pharmacological and histopathological studies have demonstrated that a 28-day exposure to CS results in a statistically significant increase in the expression levels of collagen type I (Col I) and collagen type III (Col III) in lung tissues. Hydroxyproline, a key component of collagen, was measured to quantify collagen content more accurately. During the early phase of CS exposure (day 7), no significant difference in hydroxyproline content was observed in lung tissues compared to the sham-operated group. However, by day 14 post-CS exposure, a significant increase in hydroxyproline levels was detected in the lung tissues of mice relative to the sham controls. By day 28, collagen deposition showed a progressive and pronounced increase. Notably, administration of XFBD during the initial stage of CS exposure effectively reduced and suppressed collagen accumulation, particularly in the high-dose group (Figure 4A).

To further investigate the impact of XFBD on CS-induced collagen deposition and pulmonary fibrosis, this study employed HE staining and Masson’s trichrome staining analyses on lung tissues. The HE staining results indicated that, at various time points (days 7, 14, and 28), progressive pathological changes were evident in the lung tissues of mice exposed to CS. On day 7, compared to the sham-operated group, CS-exposed mice exhibited minimal alveolar damage and slight inflammation. By day 14, lung tissues displayed significant inflammatory cell infiltration, alveolar septal thickening, and alveolar collapse. Notably, these pathological changes were substantially alleviated in the XFBD-treated groups (Figure 4B). Masson’s trichrome staining further demonstrated that collagen deposition in the lung tissues of the model group mice increased progressively with prolonged CS exposure, with pronounced fibrosis evident at later time points. In contrast, XFBD treatment significantly inhibited collagen deposition (Figure 4C).

Subsequently, Micro CT scanning was utilized to provide a comprehensive assessment of the dynamic changes in pulmonary architecture and the severity of fibrotic remodeling. In the untreated model group, there was a notable progressive increase in lung shadow attenuation from day 0 to day 14, indicating the onset and advancement of pulmonary inflammation and fibrosis. By day 28, the lung condition of the mice had significantly worsened, with the development of pulmonary parenchymal lesions characterized by the presence of ground-glass opacities in the middle and lower lung fields, as well as small nodules beneath the pleura. Notably, a 7-day treatment with XFBD at doses of 2 g/kg and 4 g/kg resulted in a substantial reduction in the diffuse high-density shadows within the lungs. Furthermore, an additional 7-day treatment with XFBD led to a further decrease in the lesion area (Figure 4D–F). Overall, the results demonstrated that XFBD exhibits a distinct protective pharmacodynamic effect during the dynamic progression of the disease, specifically during the transition from acute inflammation to fibrosis. This effect is evidenced by a notable improvement in overall lung function and a reduction in the pathological burden on the lungs.

### 2.5. XFBD Reversed EMT by Modulating Key EMT-Related Proteins

**Figure 5:**
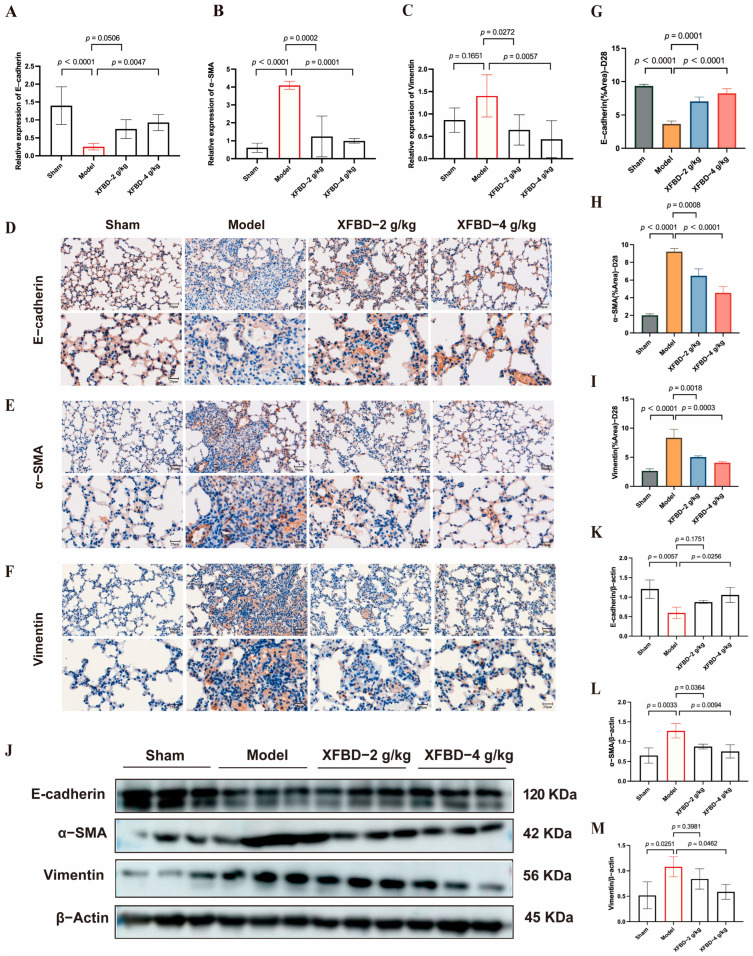
XFBD exerts a reversal effect on EMT through the regulation of critical EMT-associated proteins. (**A**–**C**) Effect of XFBD on the expression of mRNA levels of E-cadherin, α-SMA, and Vimentin in mice with silicosis. IHC analysis of paraffin-embedded, silica-induced, pulmonary fibrosis mice lung tissue sections using antibodies against E-cadherin (*n* = 5) (**D**), α-SMA (**E**), and vimentin (**F**) at 28 days (*n* = 5). Quantitative assessments of randomly selected microscopic fields from (**G**–**I**). Effect of XFBD on the expression of protein levels of E-cadherin, α-SMA, and Vimentin in mice with silicosis (**J**–**M**) (*n* = 3). Statistical analysis was performed using one-way ANOVA. Data are presented as mean ± SD.

In previous studies, we have demonstrated that XFBD can intervene in the early activation of the NLRP3 inflammasome induced by CS, thereby mitigating the inflammatory response, reducing collagen deposition, and delaying the progression of pulmonary fibrosis. Following the activation of the NLRP3 inflammasome in pulmonary fibrosis, the release of IL-1β further enhances the expression of transforming growth factor-beta 1 (TGF-β1), a pivotal factor in the initiation and progression of EMT [29]. EMT, a crucial pathological process in fibrosis, is characterized by the loss of epithelial cell polarity and the acquisition of mesenchymal cell properties. This transition increases the number of myofibroblasts, which excessively secrete extracellular matrix (ECM), leading to collagen deposition [30]. We subsequently explored the potential of XFBD to influence the expression of EMT-related proteins in order to mitigate fibrotic remodeling. Compared with the model group, the XFBD-4 g/kg treatment group exhibited a significant upregulation in the mRNA expression of E-cadherin, while the mRNA levels of Vimentin and α-smooth muscle actin (α-SMA) were significantly downregulated (Figure 5A–C). IHC analysis of paraffin-embedded, CS-induced, pulmonary fibrosis mice lung tissue sections using the antibodies against E-cadherin, α-SMA and vimentin at 28 days (Figure 5D–F). Consistent with the mRNA findings, both IHC and Western blot analyses demonstrated that XFBD significantly upregulated E-cadherin expression while downregulating Vimentin and α-SMA levels in lung tissues on day 28 (Figure 5J). These data indicate that XFBD effectively reverses EMT by modulating key EMT-related proteins, thereby contributing to its anti-fibrotic activity.

Collectively, it is evident that the role of XFBD is multifaceted rather than singular. During the initial phase of fibrosis, exemplified by day 7, XFBD primarily functions to inhibit the NLRP3 inflammasome and modulate macrophage polarization, thereby regulating the inflammatory response. As fibrosis progresses, particularly between days 14 and 28, XFBD’s role expands to include the inhibition of EMT and collagen deposition, effectively counteracting structural remodeling. This sequential and multi-stage intervention strategy allows XFBD to more effectively mitigate the progression of the disease.

## 3. Discussion

One of the most harmful environmental particulates frequently inhaled is CS. Prolonged exposure to CS is associated with an increased risk of developing cancer, interstitial fibrosis that progresses to chronic inflammation, and chronic obstructive pulmonary disease (COPD), which is characterized by airway remodeling and emphysema [31]. Due to the challenges associated with the removal of CS particles from the lungs, the present study employed CS-induced lung injury models both in vivo and in vitro to mimic the chronic lung inflammation and fibrosis. The progression of CS-induced pulmonary fibrosis follows a well-defined temporal progression, beginning with lung injury, initiated by an inflammatory response, and culminating in local repair processes. Identifying effective strategies and agents to mitigate fibrosis formation within the therapeutic window is of paramount importance [5,32]. The 28-day observation period in this study was deliberately structured to capture the critical transition from inflammation to early fibrogenesis, a crucial phase for therapeutic intervention. Our findings substantiate that exposure to CS induces significant collagen production by day 28, signifying the commencement of irreversible fibrotic commitment. While advanced fibrosis generally becomes apparent after 28 days, the molecular mechanisms driving fibrosis are unequivocally activated during this earlier period. Therefore, our approach prioritizes mechanistic insights into the early stages of disease progression, where interventions have the greatest potential to attenuate the advancement of fibrosis.

In our study, XFBD demonstrated a marked enhancement in pulmonary function during the initial phase of the disease (day 7), aligning closely with the early specific anti-inflammatory effects documented in prior in vivo and in vitro studies. These effects included inhibition of NLRP3 inflammasome activation and a reduction in the release of inflammatory mediators. XFBD’s suppression of the NLRP3 inflammasome, an early fibrotic event, decreased local inflammation and contributed to an overall enhancement in lung function. This, in turn, effectively delayed the progression of CS-induced lung injury and fibrosis. The findings of this study substantiated the hypothesis that early intervention in inflammatory responses can significantly retard the progression of fibrosis and preserve lung structural integrity. Consequently, this provided direct evidence supporting the therapeutic strategy of “early anti-inflammation to delay pulmonary fibrosis.”

Silica-induced pulmonary fibrosis is a dynamically spatiotemporal pathological process [33]. During the chronic fibrosis stage, M2 macrophages facilitate the establishment of a pro-fibrotic microenvironment [34], whereas M1 macrophages initiate the inflammatory cascade by releasing pro-inflammatory factors [35,36]. Pathological over-activation of M1 macrophages in the early stage, which is driven by NLRP3 inflammasome activation, acts as a pivotal upstream initiator of sub-sequent excessive M2 polarization and the progression of pulmonary fibrosis. In the present study, XFBD-mediated skewing of macrophage phenotype from M2 to M1 does not represent a direct promotion of M1 polarization. Instead, it functions to normalize the silica-induced pathological hyperactivation of M1 macrophages at the early stage of injury. By suppressing NLRP3 inflammasome activation and abating the excessive secretion of pro-inflammatory cytokines from over-activated M1 macrophages, XFBD weakens the upstream inflammatory signals that induce aberrant M2 polarization in the late phase, thus mitigating the formation of a pro-fibrotic microenvironment in the lung tissue. It is important to highlight that the release of the aforementioned pro-inflammatory cytokines, particularly IL-1β and IL-6, is not solely a consequence of macrophage activation. These cytokines also play a crucial role as integral components of the NLRP3 inflammasome pathway and function as positive feedback regulators. Through scavenger receptors, macrophages recognize CS particles and begin phagocytosis [37]. The invaded CS elicits an inflammatory response in macrophages, driven by the release of cytokines, such as IL-6, IL-1β, and TNF-α factors, which can induce fibrosis [38,39]. Among these pro-inflammatory cytokines, IL-6 and IL-1β are especially important. IL-6 is implicated in chronic inflammation, fibrosis, and aging. Inducing Th1 cell responses as a result of persistent inflammation, IL-6 impairs tissue repair by converting acute inflammation into a more chronic profibrotic state [40]. We also found that CS-induced LPS-primed PMs produced IL-1β, IL-6, and TNF-α in vitro, which was reversed after XFBD treatment. It is known that macrophages release IL-1β by activating the inflammasome pathway [41]. In contrast, a recent report found that IL-6 perceptibly accelerated NLRP3 inflammasome activation via the JAK2/STAT3 pathway in oral squamous cell carcinoma (OSCC) cells [42]. The above results suggest that XFBD reduces the release of pro-inflammatory factors, even under persistent CS stimulation, possibly linked with NLRP3 inflammasome activation.

Sterile and pathogen-dependent inflammation are related to the NLRP3 inflammasome [43], and its uncontrolled activation underlies several chronic inflammatory and fibrotic diseases [44]. In the progression of CS-induced pulmonary injury, NLRP3 inflammasome activation is also one of the key mechanisms [45], and NLRP3 knockout mouse models exhibit decreased aspiratory aggravation and decreased collagen deposition compared with wild-type mice following CS exposure. The NLRP3 inflammasome comprises a sensor NLRP3, an adaptor apoptosis-related speck-like protein containing a caspase recruitment domain (ASC), and an effector Caspase-1 [46]. Macrophages lacking NLRP3 or ASC failed to release cleaved IL-1β in response to CS [38]. Caspase-1 is the classical caspase involved in NLRP3/ASC inflammasome-mediated processing of IL-1β [47]. Through a two-signal process, NLRP3 inflammasome activation results in Caspase-1 activation [48]. A characteristic of Caspase-1 activation is the conversion of pro-Caspase-1 into its p20 [49]. IHC and Western blotting confirmed that XFBD greatly attenuated CS-induced NLRP3 and Cleaved-Caspase-1 expression in mice. Furthermore, XFBD remarkably reduced the number of ASC specks and suppressed the ASC oligomerization in PMs. These findings suggest that XFBD abolished the activation of CS-induced NLRP3 inflammasome by inhibiting NLRP3 inflammasome assembly.

Although our study confirmed that XFBD alleviates silica-induced silicosis and reduces NLRP3 inflammasome activation in a mouse model, the following limitations still exist, which need to be paid attention to in subsequent research. Firstly, the experimental mouse model differs substantially from clinical human silicosis. The former is established via short-term, concentrated silica instillation, which triggers rapid-onset lung inflammation and fibrosis within weeks. In contrast, human silicosis develops over years of chronic occupational silica exposure, accompanied by complex alterations in the immune microenvironment and various complications. Consequently, caution must be exercised when extrapolating the findings from animal experiments to clinical practice. Secondly, it is important to acknowledge that this study is primarily concerned with validating the pivotal role of the NLRP3 inflammasome pathway in mediating the anti-silicosis effects of XFBD. Considering that XFBD is a multi-component herbal formula, it is plausible that it may also engage other anti-inflammatory or anti-fibrotic targets. The findings of this study do not preclude the existence of additional parallel or synergistic mechanisms. Future investigations employing NLRP3 gene knockout models or cell-specific knockout techniques will be instrumental in further elucidating the necessity and contribution of this pathway within more complex in vivo environments.

Despite these limitations, our findings highlight XFBD’s promising early-intervention potential for silica-induced fibrosis, supporting its value as a candidate for preventive or adjunctive therapy in silicosis management. Notably, XFBD’s ability to suppress NLRP3-mediated inflammation at the early stage of disease progression suggests it could mitigate the irreversible fibrotic cascade before severe lung damage occurs. Future research should prioritize preclinical investigations into XFBD’s pharmacokinetic profile and long-term toxicological safety to lay a solid foundation for clinical trials. Additionally, NLRP3 activation levels and macrophage subtype ratios identified in this study could be further explored as potential biomarkers for patient stratification and real-time efficacy monitoring, facilitating the personalized application of XFBD in clinical settings.

Inflammation rapidly occurs after CS-induced lung injury, followed by collagen accumulation and fibrosis formation. Our research has demonstrated that XFBD not only significantly inhibits the early inflammatory response induced by CS during the fibrotic process but also substantially suppresses collagen deposition and fibrotic progression in the middle and late stages. Additionally, rapid persistent pulmonary inflammation upon CS stimulation triggers myofibroblast activation and abnormal extracellular matrix deposition. CS-related EMT may expand the myofibroblast pools to provide a continuous cellular source for collagen synthesis. Notably, this study found that alveolar epithelial cells undergo EMT at this stage, showing a significant spatiotemporal correlation with progressive collagen deposition. This synergy between cellular phenotypic transformation and extracellular matrix remodeling may explain the irreversible collagen deposition pattern in silicosis fibrosis. We found that XFBD reversed EMT by modulating key EMT-related proteins, specifically upregulating E-cadherin expression while downregulating Vimentin and α-SMA levels in lung tissues on day 28 post-CS exposure. We have established that XFBD can inhibit the activation of the NLRP3 inflammasome and subsequently decrease the release of IL-1β in the early stages. Given that IL-1β can enhance the expression of TGF-β1, it thereby promotes EMT [29]. It is suggested that XFBD may indirectly modulate the downstream pro-fibrotic signaling pathway of TGF-β1 and its associated EMT process through the early inhibition of NLRP3/IL-1β. This mechanism potentially elucidates the interconnected anti-inflammatory and anti-fibrotic effects of XFBD.

The occurrence and development mechanisms of pulmonary fibrosis are exceedingly intricate, encompassing the interplay of multiple cell types, molecular entities, and signaling pathways. This complexity renders the pathological process highly heterogeneous and dynamic. Consequently, drug intervention strategies targeting a single entity are insufficient to comprehensively and effectively inhibit disease progression. Therefore, there is an urgent need to transition current therapeutic paradigms towards a more integrated and multifaceted approach. The combination of anti-inflammatory and anti-fibrotic strategies represents an innovative therapeutic concept in this context. In this study, we used multiple lines of evidence in the CS-induced pulmonary fibrosis model to confirm both the anti-inflammatory and anti-fibrotic effects of XFBD. By simultaneously addressing the key processes of inflammation regulation and fibrosis progression, this dual-targeted approach can not only mitigate the inflammatory response in lung tissue and alleviate immediate patient symptoms but also effectively curb excessive collagen deposition and slow the decline in lung function, thereby achieving a more comprehensive and sustained therapeutic effect. However, more thorough and statistically sound verification of the therapeutic advantages of XFBD in patients with CS-induced pulmonary fibrosis necessitates extensive, long-term cohort clinical trials.

## 4. Materials and Methods

### 4.1. Chemicals and Reagents

XFBD was supplied by Tianjin Modern TCM Innovation Center (Batch TRT-200302). Its preparation method was described previously [50], and its chemical composition was characterized by HPLC and high-resolution mass spectrometry with molecular networking analysis [22]. XFBD is formulated with 13 medicinal herbs, including *Ephedrae herba*, *Armeniacae semen*, *Atractylodis rhizoma*, *Gypsum fibrosum*, *Coicis semen*, *Pogostemonis herba*, *Artemisiae Annuae Herba*, *Polygoni Cuspidati Rhizoma et Radix*, *Verbenae herba*, *Phragmitis rhizoma*, *Lepidii/Descurainiae semen*, *Glycyrrhizae Radix et Rhizoma*, and *Citri Grandis Exocarpium Rubrum* [51]. The mixture was soaked in distilled water at a solid–liquid ratio of 1:4 (*w*/*v*) for 30 min. The solution was heated to boiling and maintained at a rolling boil for 40 min before filtration. The filtrate was concentrated under reduced pressure to a relative density of 1.02–1.10 (measured at 60 °C) and then subjected to spray drying to obtain a dry powder for subsequent experiments [52]. Subsequently, a total of 152 components of XFBD were either fully or tentatively identified. Among these, the fully identified compounds included 44 flavonoids, 25 carboxylic acids, 23 terpenoids, 4 alkaloids, and 56 other compounds [22]. XFBD was prepared by the Tianjin Modern Traditional Chinese Medicine Innovation Center (Tianjin, China). The extract yield was 12.6%, and the total weight of the crude herbal materials used was 238 g. The equivalent dose conversion ratio between mice and humans was 0.0026:1. For clinical administration, the standard daily dose of XFBD is approximately 8.6 g/kg of body weight [22]. Based on a body weight of 20 g per mouse, the equivalent oral dose for mice was calculated as follows: 238 × 0.126 × 0.0026 ÷ 0.02 = 3.89844 g/kg, which was approximately 4.0 g/kg. This dose was designated as the high-dose group in the present study, while the low-dose group was set at half of the equivalent dose.

Silica, MTT Kits, and lipopolysaccharides were obtained from Sigma-Aldrich (St Louis, MO, USA). HE and Masson kits were purchased from Solarbio (Beijing, China). NLRP3 was purchased from Affinity Bioscience (New Jersey, USA). Cleaved-caspase1, anti-ASC, and β-actin were purchased from Cell Signaling Technology (Danvers, MA, USA). The PVDF membrane was purchased from GE Healthcare Life Science (Marlborough, MA, USA). Anti-α-Tubulin, RIPA buffer supplemented with inhibitor cocktail, and PMSF were purchased from Beyotime (Shanghai, China). Cytokines of murine IL-4 and murine IL-13 were purchased from PeproTech (Rocky Hill, NJ, USA). Mouse uncoated ELISA kits for IL-1β, TNF-α, and IL-6 were purchased from Thermo Fisher Scientific (Waltham, MA, USA).

### 4.2. Animals and Treatment

Male C57BL/6J mice (SPF, 8 weeks old, 20–22 g) were obtained from Beijing Vital River Laboratory Animal Technology Co., Ltd. (Beijing, China), and housed at the Laboratory Animal Center, Tianjin University of Traditional Chinese Medicine (Tianjin, China). During the study, all animals were cared for humanely under specific pathogen-free conditions with a controlled temperature of 22 ± 2 °C, relative humidity of 50 ± 10%, and a 12 h light/dark cycle, and had free access to water and food. Following a 7-day acclimation period, animals were randomly assigned to four groups: Sham, Model, XFBD-2 (2 g/kg), and XFBD-4 (4 g/kg). The model group and two XFBD treatment groups were injected with a 50 μL (50 mg/kg) silica suspension into the lung, once, using intratracheal injection, and the mice in the Sham group were injected with the normal saline solution. After exposure to silica for 7 d, the mice in two XFBD treatment groups were given a daily intragastric administration of 2 or 4 g/kg XFBD for 28 d. The mice in the sham and model groups were treated with saline only. For the silica-exposed group, mice that died unexpectedly from silica instillation were excluded from the analysis. No animals or data points were excluded from the control group. To reduce pain and distress to the greatest extent, all invasive operations (including intratracheal injection and intragastric gavage) were conducted gently by professionally trained researchers. The mice were monitored daily for any manifestations of discomfort to promptly address potential distress. Predicted adverse reactions mainly involved silica-induced respiratory abnormalities, whereas unforeseen events primarily consisted of occasional mortality observed in the silica-treated groups. Routine daily assessments of the animals’ general health status were also carried out throughout the experiment. The program has been approved by the Institutional Animal Care and Use Committee of the Laboratory Animal Center, Tianjin University of Traditional Chinese Medicine (Tianjin, China) (ethics approval reference number: TCM-LAEC2022267z1089, approval date: 10 August 2024).

### 4.3. Sample Size Determination and Outcome Measures

Referring to the key parameters of the pre-human silicosis model study, the sample size of 20 mice per group was determined by pre-study power analysis. The initial calculation indicated a minimum of 16 mice per group, and we adjusted to 20 to account for potential attrition and align with typical sample sizes in similar studies. Specifically, the 20 mice per group were distributed across three time points to maintain statistical robustness for analyses at each time point, while allowing longitudinal characterization of the disease process. To minimize selection bias, experimental mice were randomly assigned to control and treatment groups. The randomization sequence was generated using a computerized random number generator: each mouse was assigned a unique random value via the RANDBETWEEN function in Microsoft Excel 2021, and the mice were then evenly distributed into the respective groups based on the sorted order of these random values. This approach guaranteed that every mouse had an equal likelihood of being allocated to any experimental group. Potential contamination factors were minimized by randomly assigning cages to the control and experimental groups in the animal facility, conducting all experimental procedures in random grouping orders, and providing standardized housing and treatment conditions for all mice. No major confounders were left uncontrolled, as these strategies ensured balanced distribution of interfering factors across groups. During group allocation, only one researcher was aware of the randomization sequence to assign mice to control and treatment groups. For the conduct of the experiment, outcome assessment, and data analysis, all personnel were blinded to group allocations to avoid potential bias. The primary outcome measure, which informed the sample size calculation, was the degree of pulmonary fibrosis quantified by Micro CT.

### 4.4. Micro CT Scanning

All groups of mice were scanned on days 0, 7, and 28 after silica intratracheal injection. Animals were placed in the imaging chamber, and thoracic Micro CT acquisitions were obtained for anatomical registration and attenuation correction. Scan configuration: 90 kV tube voltage, 160 mA current, and 40 mm field width. The microfocus X-ray source (5 mm spot size) employed conical beam geometry with 68 mm maximum field width, achieving 35 μm isotropic voxel resolution. The CT scores were quantitatively analyzed according to the percentage of diffuse infiltrating shadows and dense enhancement shadow areas.

### 4.5. Histopathological Observation

The lung tissues of mice in each group were collected and made into pathological sections after embedding and fixing with 10% Neutral Formalin Fix Solution. After dehydration, they were embedded in paraffin and sectioned into 5 μm thick sections. Hematoxylin-eosin (HE) and Masson’s trichrome staining (Solarbio, Beijing, China) were used to evaluate each group’s pulmonary fibrosis degree. Under the light microscope, four visual fields were randomly selected to observe the pathological changes in each group, and the severity of the disease was judged according to the inflammatory infiltration of lung tissue, the structural integrity of alveoli, and collagen deposition.

### 4.6. Lung Function Test

The EMKA non-invasive lung function testing system was used to test the lung function of mice after 7 days, 14 days and 28 days of modeling. This included TI (inspiratory time), Te (expiratory time), TV (tidal volume), Penh (expiratory interval), and F (respiratory frequency).

### 4.7. Hydroxyproline (HYP) Detection

A HYP detection kit (Nanjing Jiancheng Bioengineering Institute, Nanjing, China) was employed to quantify hydroxyproline levels in tissue specimens. The specific procedures were as follows: Accurately weigh the tissue samples, add the hydrolysate, and adjust the pH of the mixture to neutral. Add an appropriate amount of activated carbon, centrifuge the mixture, and collect the clear, colorless supernatant. Add the reagents sequentially according to the kit instructions, incubate the mixture in a 60 °C water bath for 15 min, and then cool it to room temperature. Centrifuge the reaction mixture at 3500 rpm for 10 min, collect the supernatant, and measure the absorbance of each supernatant sample at a wavelength of 550 nm using a microplate reader (ReadMax 500F Absorbance Microplate Reader, Shanghai Shanpu Biotechnology Co., Ltd., Shanghai, China).

### 4.8. Enzyme-Linked Immunosorbent Assay

The treated cell culture supernatant of abdominal macrophages (PM) was gently aspirated with a sterile pipette and then transferred to a pre-cooled centrifuge tube. The supernatant was centrifuged at 3000 rpm at 4 °C for 10 min to remove residual cell fragments and insoluble impurities. After centrifugation, the clarified supernatants were aliquoted into sterile cryotubes and immediately stored at –80 °C; repeated freeze–thaw cycles were strictly avoided to prevent cytokine degradation. The levels of target inflammatory cytokines (IL-1β, IL-6, and TNF-α) in the processed supernatants were then quantified using commercial ELISA kits (Abcam, Cambridge, UK) in strict accordance with the manufacturer’s standardized protocols, including reagent equilibration, sequential addition of standards/samples, incubation, washing steps, and absorbance measurement at the specified wavelength.

### 4.9. Immunohistochemistry (IHC) Assay

Paraffin-embedded tissue sections were degreased with xylene, rehydrated by gradually reducing ethanol concentration, and washed with water. The sections were incubated overnight at 4 °C with the primary antibodies NLRP3 (Affinity, Changzhou, China, DF7438) and Cleaved-caspase1 (Cell Signaling Technology, Shanghai, China, 89332) and then rinsed with PBS. Following DAB development, sections were water-rinsed and hematoxylin-counterstained. Slides were mounted with neutral resin for microscopic analysis.

### 4.10. Immunofluorescent Staining

The lung sections were dewaxed, antigen-retrieved, and incubated with the first antibody F4/80 (28463-1-AP; Proteintech) in the dark at 4 °C overnight. After the second antibody was incubated and washed, sections were sealed with a mounting medium containing an anti-fluorescence quencher. For cellular immunofluorescence, slides were incubated with ASC primary antibody (Cell Signaling Technology, Shanghai, China 67824S), followed by nuclear counterstaining using a fluorescent secondary antibody. Images were acquired by fluorescence microscopy.

### 4.11. Peritoneal Macrophages Collection and Treatments

Peritoneal macrophages (PMs) were elicited as described in a previous report [53]. In brief, 1 mL of 3% thioglycolate medium (Sigma-Aldrich, St. Louis, USA, T9032) was injected intraperitoneally into the mouse’s peritoneal cavity. After 4 days, the mice were euthanized; the peritoneal cavity was gently rinsed with 5 mL of pre-cooled sterile phosphate-buffered saline (PBS), and the resulting lavage fluid (containing PMs) was collected. The washing solution was centrifuged at 1000 rpm at 4 °C for 5 min. The supernatant was discarded, and the cell pellet was resuspended in RPMI 1640 medium supplemented with 10% heat-treated fetal bovine serum and 1% penicillin/streptomycin. The cells were then seeded in culture plates and incubated; 3 h later, non-adherent cells were removed, and adherent PMs were used for further treatments.

### 4.12. Silica-Induced NLRP3 Inflammasome Activation

PMs were primed with LPS (500 ng/mL) for 5.5 h and stimulated with silica (400 μg/mL) for 4 h. For XFBD intervention, PMs were pretreated with different concentrations of XFBD (500 μg/mL, 250 μg/mL, 125 μg/mL) for 24 h. Afterward, the cell culture supernatants were collected for IL-6, TNF-α, and IL-1β ELISA. The cells were collected for ASC-speck formation and ASC oligomerization assay. PMs were incubated with ASC antibody (1:500) and a FITC-conjugated secondary antibody (1:200), respectively. The images were recorded by an ImageXpress Pico 7.1.1 (Molecular Devices, San Jose, CA, USA) and analyzed using ImageJ 1.51. For the ASC oligomerization experiment, precipitates were prepared by washing twice with PBS (1×) and crosslinking with suberic acid bis (N-hydroxysuccinimide ester) (2mM, Sigma-Aldrich, St. Louis, MO, USA).

### 4.13. Silica-Related Macrophage M2 Polarization

PMs were stimulated with IL-4 (20 ng/mL) and IL-13 (40 ng/mL) for 16 h, and silica (100 μg/mL) was added with or without XFBD (500 μg/mL). The relative expression of iNOS, YM-1, and Fizz1 was assayed by reverse transcription and quantitative PCR (RT-qPCR). RNA-Quick Purification Kit (ES Bio, Alameda, CA, USA, RN001), cDNA synthesis 5× Easy RT Master Mix (Easy-Do Bio, Hangzhou, China, PR0403050), and 2×UltraSYBR Mixture (CW Bio, Taizhou, China, CW0957) were used.

### 4.14. Western Blot Assay

Cellular proteins were isolated through lysis buffer treatment, followed by BCA protein quantification. SDS-PAGE electrophoresis was performed for protein separation before electroblotting onto PVDF membranes. The membranes underwent blocking with 5% non-fat milk in TBST solution, then were subjected to primary antibody incubation at 4 °C for 12–16 h. Finally, enhanced chemiluminescence detection was employed, and band intensity analysis was conducted with ImageJ 1.51 software.

### 4.15. Statistical Analysis

Data are reported as mean ± standard deviation (SD). Before conducting statistical tests, the assumptions of the applied tests were assessed, including normality via the Shapiro–Wilk test and homoscedasticity via Levene’s test. For comparisons among multiple independent groups, statistical analysis was performed using GraphPad Prism 10.3.0, employing one-way analysis of variance (ANOVA). For data involving two variables, repeated measures ANOVA was employed to account for within-subject correlations. A *p* value < 0.05 was considered statistically significant.

## 5. Conclusions

In summary, the study shows that XFBD can counteract CS-induced pulmonary fibrosis through several mechanisms, including inhibiting NLRP3 inflammasome activation, regulating macrophage polarization, and reversing EMT. Early in the disease (7 days post-exposure), XFBD exhibits anti-inflammatory effects by inhibiting NLRP3, reducing pro-inflammatory factors, and promoting M1 macrophage transformation. In the fibrosis stage (28 days post-exposure), it enhances E-cadherin expression, reduces Vimentin and α-SMA levels, reverses EMT, and inhibits collagen deposition and lung remodeling. Thus, XFBD shows therapeutic promise in different stages of silicosis, with its anti-inflammatory and anti-fibrotic effects contributing to its overall efficacy against CS-induced pulmonary fibrosis. This study presents experimental evidence indicating that XFBD may serve as a potential therapeutic agent for the prevention and treatment of silicosis. Furthermore, its multi-target and temporal-phase characteristics provide valuable insights for the development of traditional Chinese medicine compound strategies aimed at addressing pulmonary fibrosis.

## 6. Patents

A granted patent entitled “A Medicament for Preventing and/or Treating Silicosis and Its Application” has been obtained based on the findings of this study (Patent No. ZL202211583190.5; Applicant: Tianjin University of Traditional Chinese Medicine).

## Data Availability

The original contributions presented in this study are included in the article/Supplementary Materials. Further inquiries can be directed to the corresponding authors.

## Supplementary Materials

The following supporting information can be downloaded at: https://www.mdpi.com/article/10.3390/ph19020253/s1, Figure S1: In vitro cytotoxicity and in vivo tissue safety evaluation of XFBD.

## Abbreviations

The following abbreviations are used in this manuscript:

ASC	Apoptosis-associated speck-like protein containing caspase recruitment domain
COPD	Chronic obstructive pulmonary disease
CS	Crystalline silica
DAB	Diaminobenzidine
EMT	Epithelial–mesenchymal transition
HYP	Hydroxyproline
IHC	Immunohistochemistry
IL	Interleukin
LPS	Lipopolysaccharide
NLRP3	NOD-like receptor thermal protein domain-associated protein 3
PMs	Peritoneal macrophages
SDS-PAGE	Sulfate-polyacrylamide gel electrophoresis
TCM	Traditional Chinese Medicine
