# Supplementary material for: NLRP3 Inflammasome Inhibition by Xuanfei Baidu Decoction Attenuates Pulmonary Inflammation and Collagen Deposition in Silicosis
Source: Pharmaceuticals (Basel). 2026 Feb 1;19(2):253. doi: 10.3390/ph19020253 (PMC12943747; doi:10.3390/ph19020253)
Supplement: Supplementary file 1 [file pharmaceuticals-19-00253-s001.zip › pharmaceuticals-3994624-supplementary.pdf]

Supplementary Materials

1. Animal Ethics

All animal experiments were conducted in strict accordance with the guidelines for the care and use of laboratory animals, and the experimental protocol was approved by the Institutional Animal Care and Use Committee of the Laboratory Animal Center, Tianjin University of Traditional Chinese Medicine (Ethics Approval No.: TCM-LAEC2022267z1089; Approval Date: 10/08/2024).

2. Security detection of XFBD

The safe dose of XFBD has been verified in vitro (Supplementary Figure S1A). As a supplementary safety assessment, we also evaluated whether XFBD induces nephrotoxicity or hepatotoxicity. Morphological analysis of H&E-stained kidney and liver sections revealed no obvious pathological changes following XFBD administration (Supplementary Figure S1B).

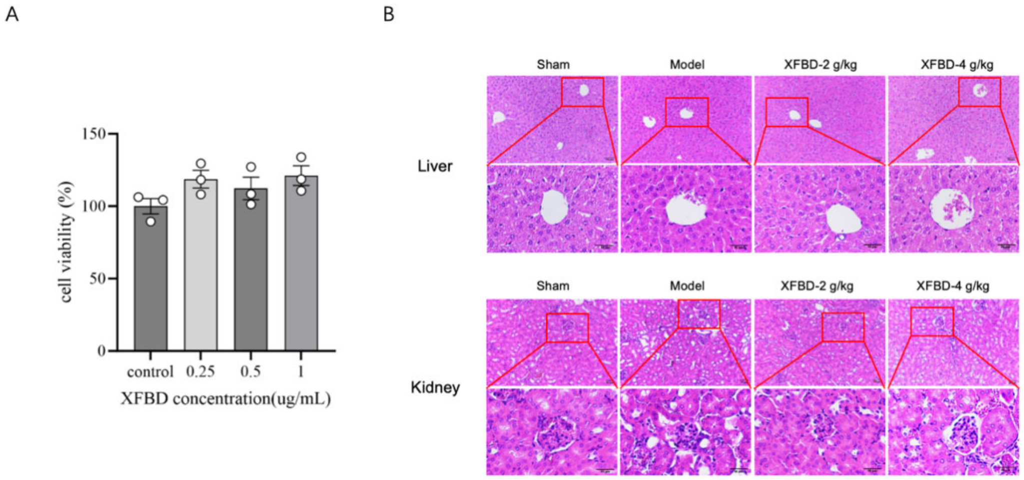

Supplementary Figure S1 In vitro cytotoxicity and in vivo tissue safety evaluation of XFBD. (A) Cell viability by MTT assay; (B) H&E staining of the liver and kidney.

3. Original Western blot image

Fig.1 C

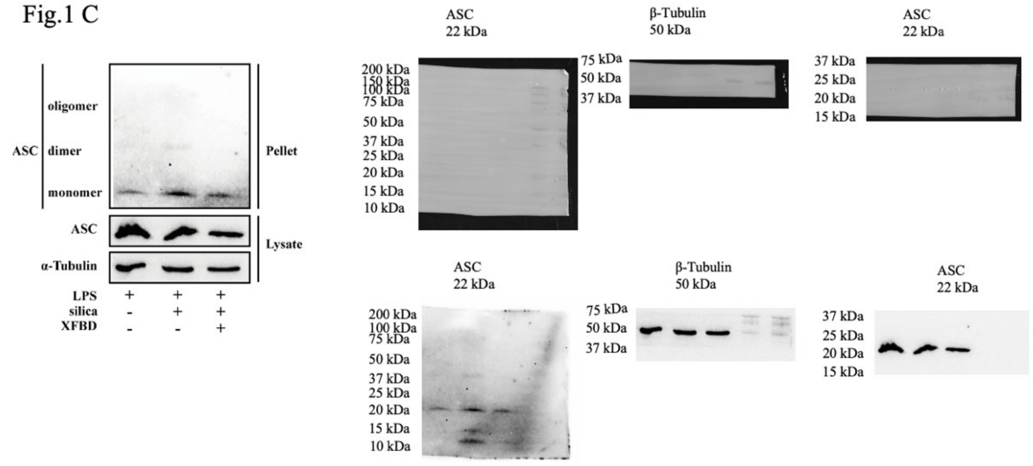

Fig.2 H

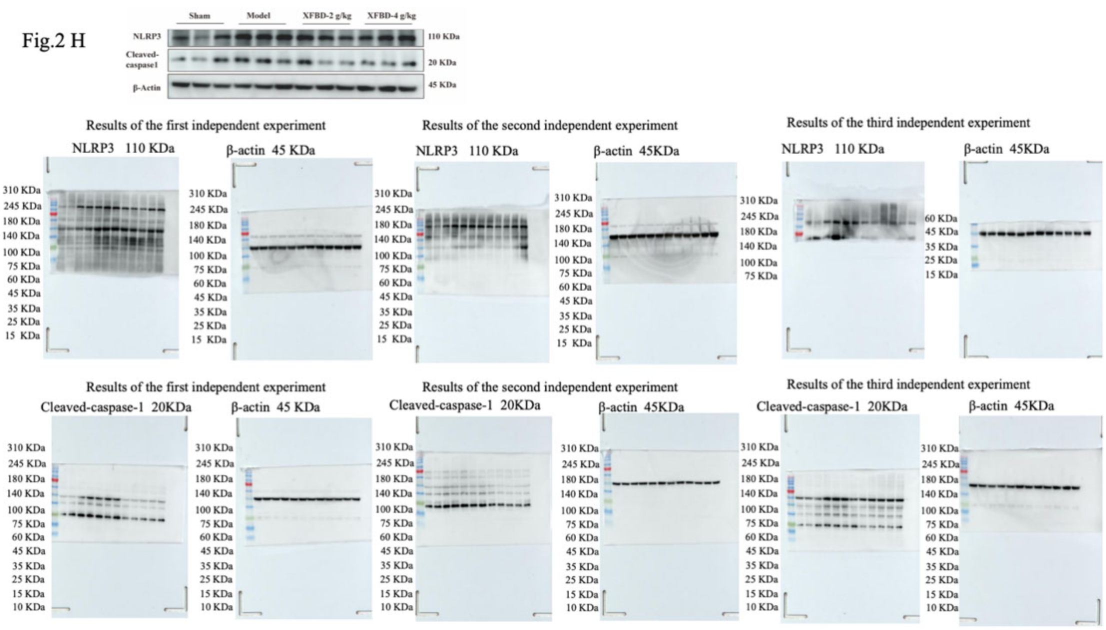

Fig.5 J

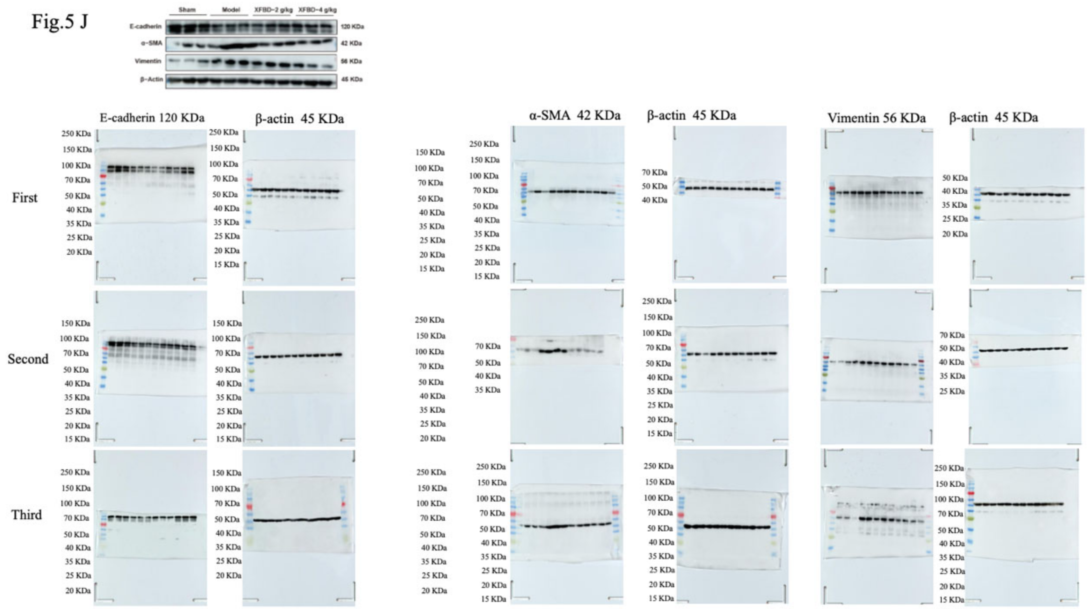
Sham
Model
XFBD-2 g/kg
XFBD-4 g/kg
E-cadherin
α-SMA
Vimentin
β-Actin
120 KDa
42 KDa
56 KDa
45 KDa
E-cadherin 120 KDa
β-actin 45 KDa
α-SMA 42 KDa
β-actin 45 KDa
Vimentin 56 KDa
β-actin 45 KDa
First
Second
Third
